# Potential Contribution of *DES* (p.Leu88Met) and *MYH7* (p.Arg787His) Variants to Familial Restrictive Cardiomyopathy

**DOI:** 10.1155/crp/6363745

**Published:** 2026-09-15

**Authors:** Mahrokh Bagherimoghaddam, Samira Kalayinia, Amir Farjam Fazelifar, Masomeh Jalilian, Sepideh Taghavi, Zahra Khajali, Majid Maleki, Ahmad Amin, Mahshid Malakootian

**Affiliations:** ^1^ Cardiogenetic Research Center, Rajaie Cardiovascular Institute, Tehran, Iran; ^2^ Cardiac Electrophysiology Research Center, Rajaie Cardiovascular Institute, Tehran, Iran; ^3^ Heart Valve Disease Research Center, Rajaie Cardiovascular Institute, Tehran, Iran; ^4^ Congenital Heart Diseases Research Center, Rajaie Cardiovascular Institute, Tehran, Iran; ^5^ Department of Cardiovascular Medicine, Mayo Clinic, Rochester, Minnesota, USA, mayo.edu

**Keywords:** *DES*, *MYH7*, RCM, restrictive cardiomyopathy

## Abstract

**Background:**

Restrictive cardiomyopathy (RCM) is a rare, severe cardiac disease with a heterogeneous genetic basis. Both genetic and nongenetic factors contribute to RCM pathogenesis. Identifying the underlying molecular causes is important for diagnosis and family screening. In this study, we investigated the genetic basis of RCM in a 52‐year‐old woman with a family history of RCM and heart disease.

**Methods:**

Genetic predisposition was evaluated using whole‐exome sequencing (WES). Candidate variants identified in the proband were validated by Sanger sequencing and interpreted using bioinformatics tools and the American College of Medical Genetics and Genomics/Association for Molecular Pathology (ACMG/AMP) guidelines.

**Results:**

Two heterozygous missense variants were identified: a novel *DES* c.262C > A (p.Leu88Met) variant and a previously reported *MYH7* c.2360G > A (p.Arg787His) variant. The *DES* variant was absent from population databases and was classified as a variant of uncertain significance, whereas the *MYH7* variant has been reported in the cardiomyopathy spectrum, primarily in hypertrophic cardiomyopathy. Although both variants may be relevant to the participant’s phenotype, their contribution remains uncertain without segregation and functional studies.

**Conclusion:**

These findings expand the spectrum of *DES* and *MYH7* variants observed in cardiomyopathy and highlight the need for further segregation and functional analyses to clarify their clinical significance in RCM. Identifying the genetic basis of RCM in this family may improve screening strategies and guide clinical management.

## 1. Introduction

Restrictive cardiomyopathy (RCM) is a rare, severe cardiac disease characterized by left atrial enlargement or biatrial enlargement, elevated atrial pressures, and normal‐sized (nondilated) or reduced ventricular diastolic and systolic volumes due to increased myocardial stiffness. In individuals with RCM, ventricular systolic function is typically preserved, whereas diastolic relaxation is impaired [1, 2]. The incidence and prevalence of RCM remain unclear and vary by geographic region, age, sex, and ethnicity [3–5]. The condition presents a broad phenotypic spectrum and is associated with a poor prognosis. Phenotypic similarities to other cardiomyopathies—including hypertrophic cardiomyopathy, dilated cardiomyopathy, and left ventricular noncompaction cardiomyopathy—along with the rarity of the disorder, have limited epidemiologic studies [1, 4].

RCM is classified as either familial or nonfamilial [6, 7]. Familial cases suggest a primary genetic cause. To date, RCM has been associated with variants in 19 genes, most of which encode sarcomeric and sarcomere‐associated proteins [7, 8]. Nonetheless, the genetic landscape of RCM needs elucidation. Advances in molecular genetics and imaging techniques have enhanced diagnostic capabilities and understanding of the underlying pathophysiology. Whole‐exome sequencing (WES) is a method employed to identify genetic causes of disease [9, 10].

The present study used WES and Sanger sequencing to investigate the genetic basis of RCM in a 4‐generation pedigree.

## 2. Methods

### 2.1. The Proband and DNA Extraction

This study included 1 participant with a diagnosis of RCM and a family history of RCM and cardiovascular disease. The study protocol was approved by the Ethics Committee of Rajaie Cardiovascular Institute (IR.RHC.REC.1399.078) and was conducted in accordance with the Declaration of Helsinki. Written informed consent was obtained from the participant. Genomic DNA was extracted from 200 µL of the proband’s peripheral blood using a commercial DNA extraction kit (DNP Kit, Iran).

### 2.2. WES, Sanger Sequencing, and Polymerase Chain Reaction (PCR)

WES was performed using the SureSelect 6 Target Enrichment Kit (Macrogen, South Korea) according to the manufacturer’s instructions. Library preparation and target enrichment were completed, and sequencing was performed on the SOLiD v4 platform (Applied Biosystems, USA) using paired‐end 2 × 150‐bp reads. The sequencing data were aligned to the human reference genome GRCh37 (hg19) using BWA. The mean depth of coverage across targeted regions was 100 ×. A total of 95% of target bases were covered at ≥ 10 ×, 90% at ≥ 20 ×, and 85% at ≥ 30 ×. Variant calling was performed using FreeBayes and SnpSift with standard quality‐control filters. Variants were retained only if they met predefined quality thresholds for mapping quality, base quality, read depth, genotype quality, and variant quality score.

Candidate variants with insufficient read support or low‐quality metrics were excluded from downstream analysis. Filtering was then applied to prioritize rare coding and splice‐region variants with a minor allele frequency of less than 0.01 in public population databases, including gnomAD, the 1000 Genomes Project, Exome Aggregation Consortium (ExAC), and Iranome. Candidate variants were assessed for predicted functional impact using multiple in silico tools and were interpreted according to the American College of Medical Genetics and Genomics/Association for Molecular Pathology (ACMG/AMP) guidelines [11]. Copy‐number variants were analyzed using ExomeDepth. Mitochondrial variants, deep intronic variants, and other noncoding splice‐impacting variants were not prioritized because WES primarily interrogates coding regions. The final candidate variants were visually inspected in the Integrative Genomics Viewer (IGV) to confirm read quality, strand balance, and the absence of obvious mapping artifacts. Sanger sequencing was used to validate the candidate variants identified by WES. The nucleotide variants identified by WES were confirmed in the proband by Sanger sequencing (3500 Genetic Analyzer, Applied Biosystems, USA). Briefly, custom‐designed primers were used to amplify genomic regions containing the target variants.

PCR amplification began with an initial denaturation at 94°C for 5 min, followed by 30 cycles of denaturation at 94°C for 30 s, gene‐specific annealing (64°C for *DES* and 59°C for *MYH7*) for 30 s, and extension at 72°C for 30 s, with a final extension at 72°C for 10 min. The PCR products were separated by electrophoresis and visualized under ultraviolet light.

Bidirectional sequencing of the *DE*S and MYH7 target regions was performed using the following primers:

DES forward: 5′‐AGGAGGGATACAAATAGTGCCG‐3′; reverse: 5′‐CTGAGAGCTGTTGCTTGGACT‐3′

MYH7 forward: 5′‐GGACATTGATCACAACCAGTACA‐3′; reverse: 5′‐GTGACTGCAGTGTGTTCATATGA‐3′

### 2.3. Bioinformatics Analysis and ACMG/AMP Variant Classification

Primer design was performed using Gene Runner (Version 6.5.50) and OligoAnalyzer. Sanger sequencing data were analyzed using BioEdit (Version 7.2.1). All detected nucleotide variants, including both known and novel variants, were examined using the UCSC Genome Browser, ClinVar, VarSome, and the Iranome database.

Variant interpretation was performed according to the ACMG/AMP guidelines for sequence variants. For each candidate variant, we systematically evaluated population frequency (gnomAD, the 1000 Genomes Project, ExAC, and Iranome), functional domain and evolutionary conservation, previous disease associations (ClinVar, VarSome, and published reports), in silico predictions (SIFT, PolyPhen‐2, PROVEAN, MutationTaster, and CADD), and the clinical context of the family. Based on these criteria, we assigned ACMG/AMP evidence codes (e.g., PM1, PM2, PS4_supporting, PP3, PP4, BP4, and BS1) and derived a final classification (pathogenic, likely pathogenic, or variant of uncertain significance [VUS]). A summary of the applied criteria for the *DES* p.Leu88Met and *MYH7* p.Arg787His variants is provided in Supporting Table 1. Protein structure analysis was performed using HOPE and UniProt.

## 3. Results

**FIGURE 1:**
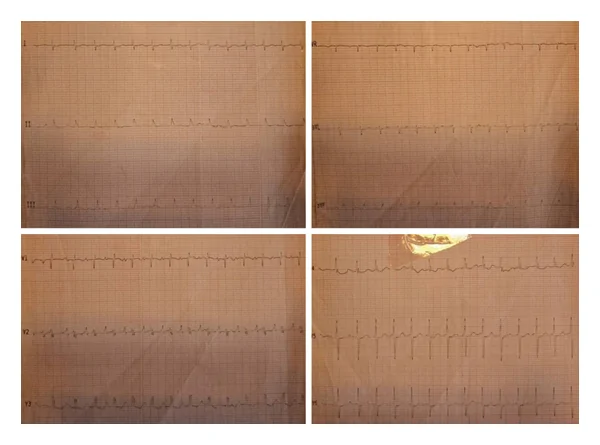
Electrocardiogram of the proband showing atrial flutter with low QRS voltage in the limb leads.

The proband was a 52‐year‐old woman (IV‐2) with a 7‐year history of cardiac symptoms who was admitted to our institute and diagnosed with RCM. Electrocardiography showed atrial flutter with low QRS voltage in the limb leads (Figure 1). Echocardiography demonstrated normal left ventricular and right ventricular size and function. Left ventricular filling pressure was elevated (E/E′, 13.7). The echocardiographic examination also showed severe biatrial enlargement (left atrial volume index, 59.6 mL/m^2^ and right atrial volume index, 36 mL/m^2^) and severe pulmonary arterial hypertension, with a systolic pulmonary artery pressure of 60–65 mm·Hg. Cardiac magnetic resonance imaging also demonstrated biatrial enlargement (left atrial area, 34 cm^2^ and right atrial area, 26 cm^2^) and subepicardial‐to‐mid‐wall fibrosis of the left ventricular lateral wall. These clinical findings, together with confirmed RCM in family members, suggested a high clinical probability of RCM. The proband had no history of hypertension or diabetes, and neither condition was considered contributory to RCM development.

**FIGURE 2:**
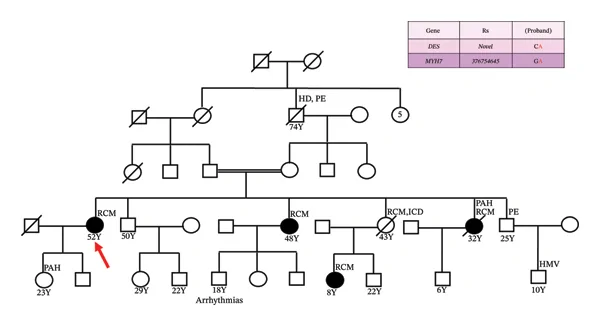
Pedigree of the proband with RCM. The arrow indicates the proband. Affected family members are shown. Abbreviations: CAD, coronary artery disease; HD, heart disease; HTN, hypertension; ICD, implantable cardioverter‐defibrillator; MVP, mitral valve prolapse; PE, pericardial effusion; RCM, restrictive cardiomyopathy.

The proband’s parents, who appeared unaffected, were consanguineously married. There was a positive family history of heart disease and RCM (Figure 2). The proband had 3 sisters and 2 brothers. One brother (IV‐3), aged 50 years, appeared unaffected, whereas the other (IV‐11) had experienced chest pain and had a history of pericardial effusion. Additionally, the 10‐year‐old son of IV‐11 (V‐11) had mitral valve prolapse.

One sister (IV‐6), aged 48 years, had also been diagnosed with RCM. Two other sisters died of RCM and heart failure and had different clinical presentations. One sister (IV‐10), aged 32 years, had a left ventricular ejection fraction greater than 50%, whereas the other sister (IV‐8), aged 43 years, had a left ventricular ejection fraction less than 35% and required device implantation (implantable cardioverter‐defibrillator). Both sisters were hospitalized at our institute and, at the time of admission, underwent echocardiography, cardiac magnetic resonance imaging, right heart catheterization, and endomyocardial biopsy. The endomyocardial biopsy confirmed idiopathic RCM.

According to the proband’s report, her maternal grandfather (II‐3) had a heart condition and experienced pericardial effusion. Her paternal grandfather (II‐1) and grandmother (II‐2) died at an early age, but the cause of death was unknown.

Furthermore, one niece (V‐8) was diagnosed with RCM at age 8 years, and another niece (V‐5), aged 18 years, had been diagnosed with arrhythmia.

### 3.1. Molecular Genetic Analysis

An unbiased WES approach was drawn upon with a mean target coverage of 100 × to identify the genetic basis of RCM in the proband. The sequencing data were analyzed using Bowtie, FreeBayes, and SnpSift for variant detection. Subsequently, the data were filtered against several population databases, including dbSNP, gnomAD v2, the 1000 Genomes Project, and Iranome.

Two heterozygous nucleotide variants were identified in the *DES* and *MYH7* genes as candidate variants. Both variants were confirmed in the proband by Sanger sequencing.

**FIGURE 3:**
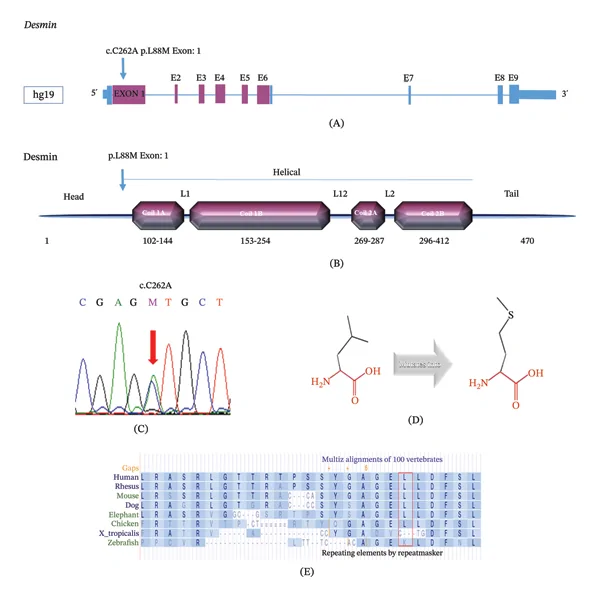
Genomic organization of the *DES* gene and domain structure of the desmin protein. (A) Genomic structure of *DES* (9 exons) showing the location of the c.262C > A variant in exon 1. (B) Domain structure of the desmin protein showing the p.Leu88Met (p.L88M) substitution in the head domain, replacing leucine with methionine. (C) Sanger sequencing chromatogram showing the heterozygous c.262C > A variant. (D) Chemical representation of the leucine‐to‐methionine substitution (p.L88M) at residue 88. (E) Conservation of the affected leucine residue across species, including humans, rhesus monkeys, mice, dogs, elephants, *Xenopus tropicalis*, and chickens.

The novel c.262C > A variant is located in exon 1 of the *DES* gene (NM_001927.4) at chr2:220283446C > A in the GRCh37 (hg19) reference genome. This variant results in substitution of leucine with methionine at amino acid position 88 (Figure 3). The heterozygous missense *DES* variant c.262C > A (p.Leu88Met) was absent from major population databases, including the 1000 Genomes Project, gnomAD, ExAC, ClinVar, and Iranome, and was located in the highly conserved head domain, which is critical for filament assembly. Nevertheless, multiple in silico prediction tools (SIFT, PolyPhen‐2, PROVEAN, and MutationTaster) predicted a tolerated, benign, or neutral effect, and no segregation or functional data were available. According to the ACMG/AMP guidelines, this variant was classified as a VUS. Still, its location in the head domain and the strong family history of cardiomyopathy provide supportive evidence for a possible contribution to the phenotype (Table 1).

**TABLE 1:** Identified variants in the *DES* and *MYH7* genes in the proband.

Gene name	Desmin (NM_001927) (NP_001918.3)	Myosin heavy chain 7 (NM_000257) (NP_000248.2)
Location (hg19)	chr2:220283446C > A	chr14:23894554C > T
Mutation variants	c.262C > A	c.2360G > A
RS	Novel	rs376754645
Amino acid changes	p.L88M	p.R787H
Genotype	Het	Het
Exon	1	21
Varsome	—	Pathogenic
CADD score	20	21.8
Sift	Tolerated	Tolerated
Polyphen 2	Benign	Possibly damaging
Mutation taster	Benign	Benign
Provean	Neutral	Neutral
1000G	Not Found	1
GnomAD	—	0.000121
Clinvar	—	Likely benign
Exac	Not Found	27
Iranome	Not Reported	0.0004160

**FIGURE 4:**
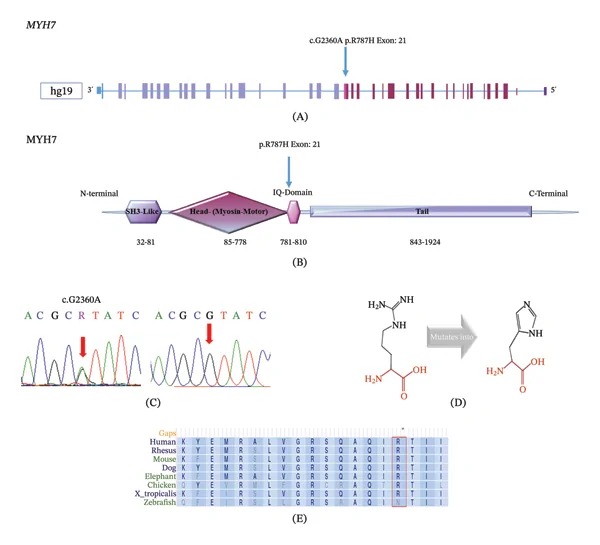
Genomic organization of the *MYH7* gene and domain structure of the myosin‐7 protein. (A) Genomic structure of *MYH7* (40 exons) showing the location of the c.2360G > A variant in exon 21. (B) Domain structure of the myosin‐7 protein showing the p.Arg787His (p.R787H) substitution in the IQ domain, replacing arginine with histidine. (C) Sanger sequencing chromatograms showing the wild‐type sequence (right) and the heterozygous c.2360G > A variant (left). (D) Schematic representation of p.R787H amino acid substitution, showing the replacement of arginine (Arg) by histidine (His). (E) Conservation of the affected arginine residue across species, including humans, rhesus monkeys, mice, dogs, elephants, chickens, *Xenopus tropicalis*, and zebrafish.

The second heterozygous variant, c.2360G > A (rs376754645), is located in exon 21 of the *MYH7* gene (NM_000257) at chr14:23894554C > T in the GRCh37 (hg19) reference genome (Figure 4). The c.2360G > A variant in *MYH7* results in an arginine‐to‐histidine substitution at amino acid position 787 (p.Arg787His). The CADD score for this variant was 21.8, and it was classified as likely pathogenic according to the ACMG/AMP guidelines. This variant has not been reported in ClinVar but is present in the 1000 Genomes Project, Iranome, ExAC, and gnomAD databases. In silico prediction tools, including PolyPhen‐2, SIFT, PROVEAN, and MutationTaster, predicted this variant to be probably damaging, tolerated, neutral, and benign, respectively (Table 1).

Given the familial nature of cardiomyopathy and the identification of *DES* and *MYH7* variants by WES in the proband, genetic counseling and clinical evaluation of affected and unaffected family members were recommended. This approach may facilitate early diagnosis of cardiac abnormalities and improve disease management.

## 4. Discussion and Conclusion

Current genetic approaches, such as linkage analysis, candidate gene analysis, family‐based studies, and next‐generation sequencing, have identified disease‐causing variants in at least 19 genes associated with RCM [4]. While these discoveries have enhanced understanding of the molecular mechanisms and potential therapeutic targets of RCM, the underlying genetic basis remains unidentified in approximately 30%–50% of idiopathic RCM cases. The boundaries of RCM are also becoming increasingly blurred because many genes associated with RCM are shared with other cardiomyopathies and cardiac phenotypes [12]. Most disease‐causing variants associated with RCM are found in genes encoding sarcomeric, cytoskeletal, or Z‐disc proteins, such as cardiac troponins, *MYH7*, *DES*, and filamin C [13–16].

Our results identified 2 candidate nucleotide variants in the *DES* and *MYH7* genes in the proband using WES. Furthermore, we identified a novel heterozygous missense variant, c.262C > A (p.Leu88Met), in the *DES* gene at chr2:220283446C > A in the GRCh37 (hg19) reference genome (Figure 3). This variant results in the substitution of leucine by methionine at amino acid position 88 of the desmin protein. In silico prediction tools classified this variant as benign or tolerated; however, it has not been reported in databases including the 1000 Genomes Project, ExAC, gnomAD, ClinVar, or Iranome. Moreover, the ACMG/AMP guidelines classify this variant as a variant of uncertain significance, and it has a CADD score of 20.

Structural analysis of this variant using HOPE [17] revealed that it is located within a stretch of residues annotated in UniProt as the head domain, which is crucial for filament assembly. The larger size of the mutant residue compared with the wild‐type residue may disrupt desmin function. The wild‐type residue is highly conserved across species, including humans, rhesus monkeys, mice, dogs, elephants, and chickens.

The desmin protein, encoded by *DES*, is a member of the intermediate filament protein family and is essential for the structural integrity of cardiac and skeletal muscle [18]. *DES* is expressed specifically in myocytes [19]. The desmin protein is composed of 3 main domains: the head, the alpha‐helical rod, and the tail. The head domain plays a critical role in proper filament assembly [19–21]. Pathogenic variants have been identified throughout the *DES* gene: those located in the C‐terminal region of coil 2 are primarily associated with skeletal muscle phenotypes, whereas variants in the head and tail domains are more commonly associated with cardiac phenotypes [18, 22].

According to previous studies [7], *DES* variants are strongly associated with RCM [23–25] and other cardiomyopathies and myopathies [22].

Most reported pathogenic variants associated with RCM are found in exons 1, 3, 6, and 8 of the *DES* gene [23–27] (Table 2). The c.46C > T (p.Thr430Ile; rs267607488) variant, previously associated with RCM, is located in exon 1 within the head domain of *DES* [23]. Although the c.262C > A (p.Leu88Met) variant has not been reported in association with RCM, *DES* variants in the head domain have been implicated in this cardiomyopathy. Consequently, it is plausible that the c.262C > A variant, which is also located in the head domain, may be associated with the RCM phenotype. Further functional studies are required to clarify the role of this variant in RCM pathogenesis.

**TABLE 2:** Reported pathogenic *DES* and *MYH7* variants associated with restrictive cardiomyopathy (RCM).

Exon	Chromosome	Mutation variants		Amino acid changes	RS	Mutation taster	Clinvar	Franklin
*Desmin (NM_001927, NP_001918.3)*								
6	chr2:220286275	c.1237G > A		P.Glu413Lys E413K	rs61726467	Disease causing	Pathogenic/Likely pathogenic	Pathogenic
1	chr2:220283230	c.46C > T		p.Arg16Cys R16C	rs60798368	Disease causing	Conflicting classifications of pathogenicity	Likely Pathogenic
8	chr2:220290454	c.1358C > T		p.Thr430Ile T453I	rs267607488	Disease causing	conflicting	Likely Pathogenic
6	chr2:220286254	c.1216C > T		p.Arg406Trp R406W	rs121913003	Disease causing	Pathogenic/Likely pathogenic	Pathogenic
1	chr2:220283548	c.364T > C		p.Tyr122His Y122H	—	Disease causing	—	Likely Pathogenic
3	chr2:220285069	c.735+1G > T		—	rs397516698	—	Pathogenic	Pathogenic
3	chr2:220285068	c.735G > C		p. Glu245Asp E245D	rs267607486	Disease causing	Pathogenic/Likely pathogenic	Likely Pathogenic

*MYH7 (NM_000257.4, NP_000248.2)*								
13	chr14:23898538		c.1157A > G	p.Tyr386Cys Y386C	rs727503269	Disease causing	Likely pathogenic	Likely pathogenic
19	chr14:23895173		c.2162G > A	p.Arg721Lys R721K	—	Disease causing	Uncertain significance	Likely pathogenic
21	chr14:23894612		c.2302G > A	p.Gly768Arg G768R	rs727503260	Disease causing	Pathogenic/Likely pathogenic	Pathogenic
21	chr14:23894566		c.2348G > A	p.Arg783His R783H	rs397516142	Polymorphism	Conflicting	Pathogenic
22	chr14:23894144		c.2513C > T	p.Pro838Leu P838L	rs397516153	Disease causing	Pathogenic	Pathogenic
22	chr14:23894139		c.2518C > A	p.Leu840Met L840M	rs730880747	Disease causing	Conflicting	Pathogenic
22	chr14:23894049		c.2608C > T	p. Arg870Cys R870C	rs138049878	Disease causing	Likely pathogenic	Pathogenic
23	chr14:23893311		c.2727C > G	p.Ile909Met I909M	rs377722048	Disease causing	Uncertain significance	Likely pathogenic
27	chr14:23889217		c.3562_3563delACinsTG	p.Thr1188Cys	—	Disease causing	—	Variant of uncertain significance

Several studies have established an association between *MYH7* variants and accelerated progression of RCM, as well as an increased likelihood of heart transplantation [16, 28, 29]. Variants in *MYH7*, which encodes a critical sarcomeric protein, are known to contribute to various cardiomyopathies, including hypertrophic cardiomyopathy and dilated cardiomyopathy [30, 31]. Furthermore, pathogenic and likely pathogenic *MYH7* variants have been documented in both hypertrophic cardiomyopathy and RCM, and these variants are frequently associated with atrial fibrillation and an increased risk of heart failure [16, 32].

Multiple investigations have identified specific disease‐causing *MYH7* variants associated with RCM. These include rs727503269 [33], rs727503260 [28, 29], rs397516142 [34], rs730880747 [28], rs377722048 [28], rs397516153 [34], and rs138049878 [35], as well as the coding variants c.2162G > A (p.Arg721Lys) [36] and c.3562_3563delACinsTG (p.Thr1188Cys) [8, 37] (Table 2).

In this study, we identified the c.2360G > A (rs376754645) variant in the *MYH7* gene. This variant is located in exon 21 and resides within the neck/hinge domain of the myosin‐7 protein. It results in an arginine‐to‐histidine substitution at amino acid position 787 (p.Arg787His). This region is known to interact with the myosin essential light chain [38] and is highly conserved across species, including humans, rhesus monkeys, mice, dogs, elephants, chickens, and *Xenopus tropicalis* (Figure 4).

Kostareva et al. [28] and Ware et al. [29] described the c.2302G > A (p.Gly768Arg; rs727503260) and c.2348G > A (p.Arg783His; rs397516142) *MYH7* variants in exon 21 as pathogenic and likely pathogenic, respectively, in association with RCM. According to HOPE [17], the mutant residue is smaller and has a neutral charge compared with the wild‐type residue. Previous reports have suggested that variants in regions involved in protein–protein interactions may disrupt protein function [39]. The c.2360G > A (p.Arg787His) *MYH7* variant has been reported as a pathogenic variant associated with hypertrophic cardiomyopathy [39–41].

In 2006, Laredo et al. [41] identified this variant in conjunction with the p.Ile736Thr variant in a woman with severe left ventricular hypertrophy. Additionally, Purushotham et al. [39] reported the c.2360G > A (p.Arg787His) *MYH7* variant as pathogenic in 2 unrelated families with hypertrophic cardiomyopathy in 2010. To our knowledge, this is the first report of this variant as a potential contributor to the RCM phenotype. Further functional analyses are necessary to clarify the role of this variant in RCM.

Pedigree analysis of the proband indicates a familial pattern of RCM. Her parents are first cousins, and she has multiple family members affected by RCM or related cardiovascular conditions. Two of her sisters died of RCM and heart failure, and a third sister (aged 48 years) has been diagnosed with RCM. One brother has a history of pericardial effusion, and a niece (whose mother died at age 43 years after requiring an implantable cardioverter‐defibrillator) has also been diagnosed with RCM. This distribution of affected individuals within the family suggests a genetic basis for RCM in this pedigree. Unfortunately, DNA from the proband’s family members was unavailable for genetic and segregation analyses. The coexistence of the 2 identified variants in the proband, either together or independently, may contribute to the development of RCM and potentially indicate a digenic inheritance model. Although *DES* and *MYH7* are well‐established cardiomyopathy genes, the evidence supporting the 2 variants identified in this family is not equivalent. The novel *DES* c.262C > A (p.Leu88Met) variant has no prior disease reports, and its interpretation relies primarily on indirect evidence, such as gene relevance, domain location, evolutionary conservation, and the phenotype of the proband. In contrast, the *MYH7* c.2360G > A (p.Arg787His) variant has been reported previously but primarily in cases of hypertrophic cardiomyopathy rather than RCM. Importantly, segregation analysis was unavailable, and computational predictions were not fully concordant for either variant. Therefore, the possibility that 1 or both variants are incidental findings cannot be excluded. Accordingly, the proposed digenic model remains a hypothesis pending segregation and functional validation.

Although *DES* and *MYH7* variants are typically inherited in an autosomal dominant manner, the unaffected parents in this pedigree could have carried a single heterozygous variant with incomplete penetrance. Modifier factors, such as environmental influences or protective alleles, could suppress phenotypic expression. The occurrence of RCM in the siblings suggests a shared genetic etiology; nonetheless, the lack of parental genotyping data renders the digenic inheritance hypothesis speculative. Additional genetic testing and functional analyses are needed to evaluate this hypothesis and clarify the clinical significance of these variants.

Importantly, the *MYH7* c.2360G > A (p.Arg787His; rs376754645) variant is present at low allele frequencies in several population databases, including gnomAD, ExAC, the 1000 Genomes Project, and Iranome. Even though these frequencies are consistent with a rare variant in a cardiomyopathy‐associated gene, they argue against a fully penetrant Mendelian cause of RCM in the general population. Instead, they support a model of reduced penetrance and variable expressivity, which has been described in *MYH7*‐associated hypertrophic and dilated cardiomyopathies, in which pathogenic or potentially pathogenic variants may be identified in apparently unaffected individuals. The nonzero population frequency, together with database annotations such as “likely benign” in ClinVar, underscores phenotype‐specific uncertainty and suggests that the *MYH7* p.Arg787His variant is more likely to represent a cardiomyopathy‐associated variant with incomplete penetrance and possible modifier effects than a single causative variant for familial RCM in this pedigree.

In conclusion, our findings are consistent with previous studies highlighting the importance of identifying novel nucleotide variants associated with RCM, a rare disease with a poor prognosis and limited pharmacologic treatment options. Identification of such variants may inform future research on disease mechanisms, variant interpretation, and potential therapeutic strategies.

## 5. Limitations of the Study

Several limitations should be considered when interpreting the results of this study. The inability to perform segregation analysis because of the unavailability of DNA samples from affected and unaffected family members limited our ability to evaluate the contribution of the identified variants to the disease. Furthermore, the study did not include functional studies, protein analyses, or gene expression analyses, which are essential for elucidating the roles of these variants in RCM pathogenesis. Future investigations incorporating these approaches are needed to clarify the roles and clinical significance of these variants in RCM.

## Author Contributions

• Mahrokh Bagherimoghaddam: performed laboratory work, generated data, and edited the manuscript.

• Masomeh Jalilian: performed laboratory work, generated data, and edited the manuscript.

• Samira Kalayinia: performed WES analysis and edited the manuscript.

• Amir Farjam Fazelifar: performed clinical evaluations and edited the manuscript.

• Sepideh Taghavi: performed clinical evaluations and edited the manuscript.

• Zahra Khajali: performed clinical evaluations, drafted the manuscript, and edited the final version.

• Majid Maleki: performed clinical evaluations and edited the manuscript.

• Ahmad Amin: performed clinical evaluations and edited the final manuscript.

• Mahshid Malakootian: conceived and designed the study, generated and interpreted the data, drafted the manuscript, and edited the final version.

## Funding

This study was funded by the Research Deputyship of Rajaie Cardiovascular Institute (Grant No. 99057) and awarded to Dr Malakootian and Dr Amin.

## Disclosure

All authors contributed to the manuscript and approved the final version.

## Ethics Statement

The Ethics Committee of Rajaie Cardiovascular Institute approved the study protocol (IR.RHC.REC.1399.078), which was conducted in accordance with the Declaration of Helsinki. The proband provided written informed consent.

## Consent

This study does not contain identifiable individual information.

## Conflicts of Interest

The authors declare no conflicts of interest.

## Supporting Information

Additional supporting information can be found online in the Supporting Information section.

## Supporting information


**Supporting Information** ACMG/AMP evidence codes and final classification for the *DES* p.Leu88Met and *MYH7* p.Arg787His variants identified in the proband.

## Data Availability

The variant reported in this study is available under accession number rs376754645. All datasets were deposited in online repositories. Variant analysis was performed using the UCSC Genome Browser and ClinVar databases. Pathogenicity assessment of the identified variants was conducted using multiple in silico prediction tools: • Combined Annotation Dependent Depletion (CADD; Phred score > 15). • SIFT. • PROVEAN. • PolyPhen‐2. • MutationTaster. • HOPE protein analysis tool. Complete accession numbers and repository information are provided in the main article and the Declarations section.
