# Supplementary material for: Potential Contribution of DES (p.Leu88Met) and MYH7 (p.Arg787His) Variants to Familial Restrictive Cardiomyopathy
Source: Cardiol Res Pract. 2026 Sep 15;2026:6363745. doi: 10.1155/crp/6363745 (PMC13576896; doi:10.1155/crp/6363745)
Supplement: Supplementary file 1 [file CRP-2026-6363745-s001.docx]

**Supplementary Table 1.** ACMG/AMP evidence codes and final classification for the *DES* p.Leu88Met and *MYH7* p.Arg787His variants identified in the proband.

| **Variant** | **Gene** | **ACMG/AMP criteria**  **(with evidence)** | **Final classification** |
| --- | --- | --- | --- |
| c.262C>A (p.Leu88Met), chr2:220283446 C>A, NM_001927.4 | *DES* | PM2 (absent from gnomAD, 1000 Genomes, ExAC, Iranome); PM1_supporting (located in the conserved head domain, crucial for filament assembly); PP4 (family history consistent with inherited cardiomyopathy); BP4 (multiple in silico tools indicate tolerated/benign effect) | Variant of uncertain significance (VUS) |
| c.2360G>A (p.Arg787His), chr14:23894554 C>T, NM_000257.4 | *MYH7* | PM1 (neck/hinge/IQ domain interacting with myosin essential light chain); PM2 (very low allele frequency in gnomAD/ExAC); PS4_supporting/PP5 (previous reports of the same variant in families with hypertrophic cardiomyopathy); PP3 (predominantly damaging in silico predictions); BS1 (present at very low frequency in population databases) | Likely pathogenic (cardiomyopathy spectrum, including RCM) |
